# Pre-procedural Serum Lipid Profile and Post-procedural Myocardial Injury

**DOI:** 10.5812/cardiovascmed.14739

**Published:** 2013-10-28

**Authors:** Hamidreza Sanati

**Affiliations:** 1 Cardiovascular Intervention Research Center, Rajaie Cardiovascular, Medical and Research Center, Iran University of Medical Sciences, Tehran, IR Iran

**Keywords:** Percutaneous coronary intervention, Dyslipidemias

Percutaneous coronary intervention (PCI) has the ability to relieve symptoms in chronic ischemic heart disease and might change the natural history of acute coronary syndromes. However, it could be potentially hazardous and result in adverse procedural outcomes. Complications might occur despite the application of the best management practices and frequently result in unsatisfactory clinical outcomes. Periprocedural myocardial infarction (MI) is one of the most common complications of PCI (1). In clinical practice, asymptomatic creatine kinase-MB (CK-MB) elevations < 5 times the upper limit of the normal range (ULN) occur after 3% to 11% of technically successful PCIs and have little apparent clinical consequence. Larger degrees of myonecrosis (CK-MB ≥ 5 times ULN) predict higher one -year mortality rates (2). Now in the journal, Maadani et al. reported the relationship between the pre-procedural serum lipid profile and post-procedural myocardial injury in patients who have undergone elective PCI (3). They have studied 138 patients without evidence of preprocedural MI according to the normal values of CK.MB. The incidence of post-procedural MI was about 25% (35 patients) which is very high and quite different from recent studies (4). It seems that the authors have evaluated post-PCI myonecrosis defined as CK-MB elevation ≥ ULN after the procedure but not the periprocedural infarction which is Ck.MB exceeding three times more than ULN according to the universal definitions of MI (5). There are several mechanisms which could be responsible for myonecrosis after the procedure (6-8). Apparent procedural complications such as stent thrombosis, coronary dissection, and occlusion of the large side branches have not been excluded from the study or at least not cited in the article. The occurrence of such complications can result in selection bias. There are no data regarding the angiographic and procedural characteristics of the patients; and many other causes that act as the confounding factors have not been addressed in the study and adjusted in the analytic process. There was no significant difference in lipid profiles between the patients with and without myonecrosis. This finding could be predictable because of the small number of studied patients and the fact that mechanical causes of post-procedural myonecrosis have not been excluded. Dyslipidemia serves as a major risk factor for coronary events but how can we interpret its effects into the myocardial injury after PCI with a wide range of causative factors?

## References

[1] Bhatt DL, Topol EJ (2005). Does creatinine kinase-MB elevation after percutaneous coronary intervention predict outcomes in 2005? Periprocedural cardiac enzyme elevation predicts adverse outcomes.. Circulation..

[2] Prasad A, Gersh BJ, Bertrand ME, Lincoff AM, Moses JW, Ohman EM (2009). Prognostic significance of periprocedural versus spontaneously occurring myocardial infarction after percutaneous coronary intervention in patients with acute coronary syndromes: an analysis from the ACUITY (Acute Catheterization and Urgent Intervention Triage Strategy) trial.. J Am Coll Cardiol..

[3] Maadani M, Abdi S, Parchami-Ghazaee S, Alizadeh K, Fathi H, Musavi R (2013). Relationship between Pre-Procedural Serum Lipid Profile and Post- Procedural Myocardial Injury in Patients Undergoing Elective Percutaneous Coronary Intervention. Res Cardiovasc Med.

[4] Muschat X, Slimani A, Jamart J, Chenu P, Dangoisse V, Gabriel L (2012). The different mechanism of periprocedural myocardial infarction and their impact on in-hospital outcome.. J Invasive Cardiol ..

[5] Thygesen K, Alpert JS, White HD, Jaffe AS, Apple FS, Galvani M (2007). Universal definition of myocardial infarction.. Circulation..

[6] Bonderman D, Teml A, Jakowitsch J, Adlbrecht C, Gyongyosi M, Sperker W (2002). Coronary no-reflow is caused by shedding of active tissue factor from dissected atherosclerotic plaque.. Blood..

[7] Saber RS, Edwards WD, Bailey KR, McGovern TW, Schwartz RS, Holmes DR, Jr Coronary embolization after balloon angioplasty or thrombolytic therapy: an autopsy study of 32 cases.. J Am Coll Cardiol..

[8] Topol EJ, Yadav JS (2000). Recognition of the importance of embolization in atherosclerotic vascular disease.. Circulation..

